# Association between LRP1 C766T polymorphism and Alzheimer’s disease susceptibility: a meta-analysis

**DOI:** 10.1038/s41598-017-08335-w

**Published:** 2017-08-16

**Authors:** Yun Wang, Shengyuan Liu, Jingjing Wang, Jie Zhang, Yaqiong Hua, Hua Li, Huibiao Tan, Bin Kuai, Biao Wang, Sitong Sheng

**Affiliations:** 1 College of Life Sciences and Oceanography, Shenzhen University, Shenzhen, 518060 China; 2 Department of Chronic Noncommunicable Disease Control, Shenzhen Nanshan Center for Chronic Disease Control, Shenzhen, 518054 China; 3 HYK High-throughput Biotechnology Institute, Shenzhen, 518057 China

## Abstract

Low density lipoprotein receptor-related protein 1 (LRP1) C766T polymorphism (rs1799986) has been extensively investigated for Alzheimer’s disease (AD) susceptibility. However, results in different studies have been contradictory. Therefore, we conducted a meta-analysis containing 6455 AD cases and 6304 controls from 26 independent case–control studies to determine whether there was an association between the LRP1 C766T polymorphism and AD susceptibility. The combined analysis showed that there was no significant association between LRP1 C766T polymorphism and AD susceptibility (TT + CT versus CC: OR = 0.920, 95% CI = 0.817–1.037, *P* = 0.172). In subgroup analysis, significant decreased AD susceptibility was found among Asian population in allele model (T versus C: OR = 0.786, 95% CI = 0.635–0.974, *P* = 0.028) and dominant model (TT + CT versus CC: OR = 0.800, 95% CI = 0.647–0.990, *P* = 0.040). Moreover, T allele of LRP1 C766T was statistically associated with late onset of AD (LOAD) (T versus C: OR = 0.858, 95% CI = 0.748–0.985, *P* = 0.029; TT + CT versus CC: OR = 0.871, 95% CI = 0.763–0.994, *P* = 0.040). In conclusion, our meta-analysis suggested that LRP1 C766T polymorphism was associated with lower risk of AD in Asian, and could reduce LOAD risk especially. Considering some limitations of our meta-analysis, further large-scale studies should be done to reach a more comprehensive understanding.

## Introduction

Alzheimer’s disease (AD), a progressive and lethal neurodegenerative disorder, has become a global challenge for the 21st century^1, 2^. It is essentially characterised by cerebral senile plaques laden with β-amyloid peptide (Aβ), dystrophic neurites in neocortical terminal fields as well as neurofibrillary tangles of hyperphosphorylated microtubule-associated protein tau^3^. Besides, loss of neurons and white matter, congophilic angiopathy, inflammation, and oxidative damage are also important pathological features of AD. It is believed that genetic factors, lifestyle and environmental factors synergistically give rise to AD. Variants associated with AD have been detected in more than 20 genes, which are involved in metabolism, inflammation, synaptic activity and intracellular trafficking^4, 5^.

Low density lipoprotein receptor-related protein 1 (LRP1) has been widely studied due to its pleiotropic roles in AD pathogenesis^6^. LRP1 is ubiquitously expressed in various tissues, especially high in liver, lung and brain^7^. In the central nervous system, LRP1 plays an important role in controlling Aβ metabolism and maintaining brain homeostasis. There are two forms of LRP1–soluble LRP1 and cell-surface LRP1. In plasma, soluble LRP1 binds to peripheral Aβ, and consequently prevents free Aβ access to the brain^8^. As a cell surface receptor, LRP1 can control the endocytosis of multiple ligands, mediate cell signaling transductions and regulate gene expression through its intracellular domain^9–11^. For instance, the interaction between amyloid precursor protein (APP) and cell-surface LRP1 leads to increased endosomal trafficking of APP, accelerating Aβ production. Besides that, Aβ can enter multiple cell types (eg. abluminal brain endothelial cell and hepatic cell) through cell-surface LRP1, in which the ubiquitous apolipoprotein E (APOE) and activated alpha-2-macroglobulins (A2M) are chaperones, and subsequently degraded by endopeptidase^12^. Therefore, LRP1 are involved in the bulk transport, primary production, brain and systemic clearance of AD toxin Aβ, and thus plays a critical role in AD pathogenesis.

The silent C766T polymorphism in exon 3 of LRP1 gene (rs1799986) has attracted extensive attention since first reported as a risk factor for AD^13^. However, results in different studies have been contradictory. The inconsistency is likely to relate with insufficient statistical power, racial differences or other demographic variables. Therefore, we conducted a comprehensive meta-analysis to determine whether there was an association between the LRP1 C766T polymorphism and AD susceptibility.

## Results

### Eligible studies

**Figure 1:**
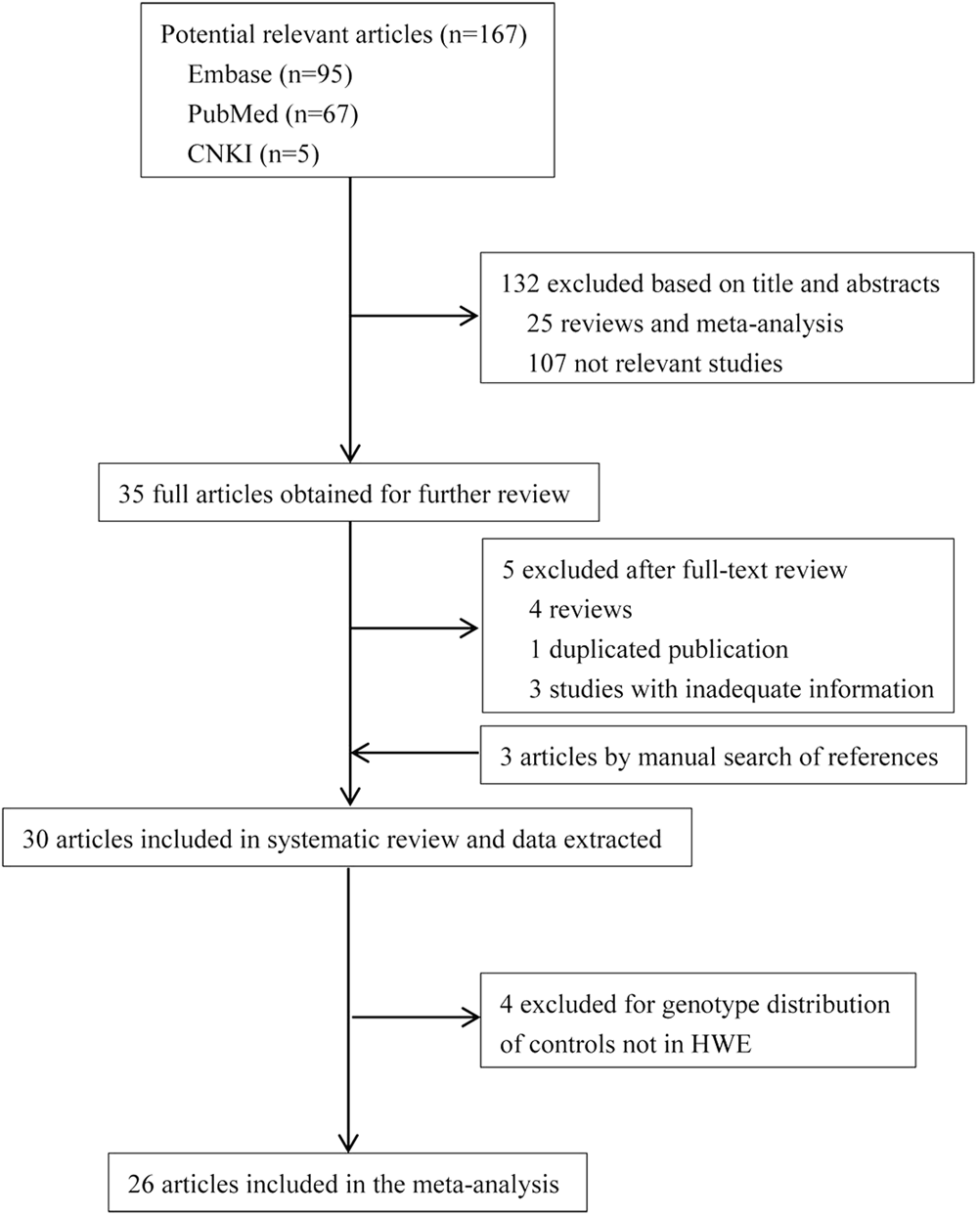
Flow chart of selection studies in our meta-analysis.

A total of 167 relevant studies were identified from initial database searching, of which 35 publications were included based on titles and abstracts (Fig. 1). Furthermore, 4 reviews, 1 duplicated publication and 3 studies with inadequate information were excluded after careful reading of the full text. Besides, manual search of references revealed 3 more articles. After primary data extracted from the 30 independent studies, 4 studies were excluded for genotype distribution of controls was not in Hardy-Weinberg equilibrium﻿ (HWE)^14–17^. Finally, 26 eligible studies containing 6455 AD cases and 6304 controls were included in our meta-analysis. The characteristics of the 26 studies on LRP1 C766T polymorphism and AD susceptibility was summarized in Table 1. The ethnicities of these subjects involved in the comparisons were diverse, including Caucasian (n = 16), Asian (n = 6), African (n = 1) and mixed (n = 3). Besides, LRP1 C766T genotype and allele distribution among AD cases and controls was summarized in Table 2, and the control group in all studies was in HWE.

**Table 1:** Characteristics of individual studies included in the meta-analysis.

First author	Year	Country	Ethnicity	AD				Controls			Criteria for AD diagnosis	Genotyping method	Source of control	Time of AD onset	Quality score
				N^a^	Age^b^	Age^c^	Gender^d^	N	Age^b^	Gender^d^					
Yuan, Q.^50^	2013	China	Asian	364	74.9	69.9	57%	291	73.7	60%	NINCDS-ADRDA	PCR and Direct sequencing	HB	Mixed	9
Vargas, T.^32^	2010	Spain	Caucasian	746	NA	73.7	66%	598	74.8	68%	NINCDS-ADRDA and DSM-IV	TaqMan SNP Genotyping Assays	PB	NA	12
Vazquez-Higuera, J. L.^52^	2009	Spain	Caucasian	246	76.6	72.9	65%	237	81.2	69%	NINCDS-ADRDA	PCR-RFLP	PB	Mixed	10
Chen, Y.^29^	2009	China	Asian	67	71.9	NA	34%	77	70.0	45%	NINCDS-ADRDA	PCR-RFLP	PB	NA	8
Bahia, V. S.^33^	2008	Brazil	Mixed	120	75.2	71.2	68%	120	72.5	63%	NINCDS-ADRDA and DSM-IV	PCR-RFLP	PB	Mixed	10
Rodriguez, E.^34^	2006	Spain	Caucasian	274	75.4	71.6	68%	283	80.5	71%	NINCDS-ADRDA	PCR-RFLP	PB	Mixed	8
Forero, D. A.^35^	2006	Colombia	Mixed	106	73.3	68.8	71%	97	72.2	NA	NINCDS-ADRDA	PCR-RFLP	NA	Mixed	7
Pritchard, A-1^36^	2005	UK	Caucasian	250	NA	56.7	55%	235	50.9	52%	NINCDS-ADRDA and DSM-III-R	PCR-RFLP	PB	Early	9
Pritchard, A-2^36^	2005	UK	Caucasian	183	NA	73.8	65%	220	76.8	44%	NINCDS-ADRDA and DSM-III-R	PCR-RFLP	PB	Late	9
Bian, L.^60^	2005	China	Asian	216	NA	74.7	NA	200	72.0	NA	NINCDS-ADRDA and DSM-IV	PCR-RFLP	PB	Late	11
Panza, F.^37^	2004	Italy	Caucasian	166	69.4	NA	62%	225	71.3	68%	NINCDS-ADRDA	Roche LightCycler Genotyping	PB	Mixed	9
Zheng, W. D.^38^	2004	China	Asian	79	72.8	>65	49%	156	71.2	41%	NINCDS-ADRDA	PCR-RFLP	PB	Late	10
Kolsch, H.^31^	2003	Germany	Caucasian	212	73.1	NA	71%	337	73.2	61%	DSM-IV	PCR-RFLP	PB + HB	NA	12
Helbecque, N-1^53^	2003	France	Caucasian	239	74.0	NA	65%	232	79.0	68%	NINCDS-ADRDA and DSM-III-R	PCR-RFLP	HB	NA	10
Helbecque, N-2^53^	2003	France	Caucasian	56	85.0	NA	80%	180	79.0	51%	NINCDS-ADRDA and DSM-III-R	PCR-RFLP	HB	NA	9
Perry, R. T.^39^	2001	USA	African	111	71.3	NA	78%	78	75.2	76%	NINCDS-ADRDA	PCR-RFLP	PB	NA	11
Bi, S.^28^	2001	China	Asian	38	70.2	NA	45%	40	69.2	40%	NINCDS-ADRDA	PCR-RFLP	PB	NA	8
Sanchez-Guerra, M.^40^	2001	Spain	Caucasian	305	75.5	71.8	68%	304	80.4	72%	NINCDS-ADRDA	PCR-RFLP	PB	Mixed	12
McIlroy, S. P.^41^	2001	UK	Caucasian	219	77.5	>65	67%	237	77.2	70%	NINCDS-ADRDA and DSM-IV	PCR-SSCP	PB	Late	12
Prince, J. A.^42^	2001	Sweden	Caucasian	204	NA	NA	61%	171	NA	63%	NINCDS-ADRDA	PCR-SSCP	PB + HB	Late	10
Verpillat, P.^43^	2001	France	Caucasian	274	NA	65.5	56%	290	67.4	57%	NINCDS-ADRDA	PCR-RFLP	PB	NA	12
Bullido, M. J.^51^	2000	Spain	Caucasian	199	NA	70.4	60%	243	72.0	62%	NINCDS-ADRDA	PCR-SSCP	PB	Late	10
Hatanaka, Y.^48^	2000	Japan	Asian	100	NA	76.6	68%	246	79.4	NA	NINCDS-ADRDA and DSM-IV	PCR-RFLP	PB	Late	8
Bertram, L.^45^	2000	USA	Mixed	276	NA	71.7	NA	194	NA	NA	NINCDS-ADRDA	PCR-SSCP	PB	NA	11
Beffert, U.^44^	1999	Canada	Caucasian	225	NA	70.9	48%	187	NA	41%	NA	PCR-RFLP	PB + HB	NA	9
Kamboh, M. I.^49^	1998	USA	Caucasian	432	75.4	68.6	62%	106	67.8	59%	NINCDS-ADRDA and DSM-III-R	PCR-SSCP	NA	NA	9
Lambert, J. -C.^30^	1998	France	Caucasian	558	71.8	68.6	62%	596	72.7	63%	NINCDS-ADRDA and DSM-III-R	PCR-SSCP	NA	NA	9
Kang, D. E.^13^	1997	USA	Caucasian	157	>65	73.2	53%	102	77.1	53%	NINCDS-ADRDA	PCR-SSCP	PB	Late	11

NINCDS: the National Institute of Neurological Disorders and Stoke; ADRDA: Alzheimer Diseases and Related Disorders Association; DSM: the Diagnostic and Statistical Manual of Mental Disorders; NA: not available; PB: population-based control; HB: hospital-based control. ^a^Number. ^b^Age at survey. ^c^Age at onset of Alzheimer’s disease. ^d^Percentage of female.

**Table 2:** LRP1 C766T genotype and allele distribution among AD cases and controls in the included studies.

First author	AD					Control					HWE
	CC	CT	TT	C	T	CC	CT	TT	C	T	*P* ^a^
Yuan, Q.^50^	304	54	6	662	66	232	52	7	516	66	0.058
Vargas, T.^32^	559	172	15	1290	202	442	138	18	1022	174	0.079
Vazquez-Higuera, J. L.^52^	193	51	2	437	55	198	35	4	431	43	0.107
Chen, Y.^29^	59	8	0	126	8	56	19	2	131	23	0.800
Bahia, V. S.^33^	87	28	5	202	38	86	30	4	202	38	0.497
Rodriguez, E.^34^	211	NA	NA	NA	NA	233	NA	NA	NA	NA	0.576
Forero, D.A.^35^	84	22	0	190	22	78	18	1	174	20	0.972
Pritchard, A.^36^	337	115	14	789	143	334	132	11	800	154	0.629
Bian, L.^60^	189	26	1	404	28	179	21	0	379	21	0.433
Panza F^37^	115	49	2	279	53	160	63	2	383	67	0.116
Zheng, W. D.^35^	72	6	1	150	8	139	16	1	294	18	0.478
Kolsch, H.^31^	145	59	8	349	75	250	84	3	584	90	0.156
Helbecque, N.^53^	216	70	9	502	88	290	108	14	688	136	0.321
Perry, R. T.^39^	97	14	0	208	14	74	4	0	152	4	0.816
Bi, S.^28^	31	6	1	68	8	24	13	3	61	19	0.516
Sanchez-Guerra, M.^40^	237	65	3	539	71	249	51	4	549	59	0.457
McIlroy, S. P.^41^	193	24	2	410	28	198	37	2	433	41	0.852
Prince, J. A.^42^	155	47	2	357	51	124	41	6	289	53	0.269
Verpillat, P.^43^	198	71	5	467	81	214	66	10	494	86	0.092
Bullido, M. J.^51^	151	47	1	349	49	173	66	4	412	74	0.417
Hatanaka, Y.^48^	83	17	0	183	17	200	45	1	445	47	0.358
Bertram, L.^45^	186	82	8	454	98	135	55	4	325	63	0.556
Beffert, U.^44^	158	58	9	374	76	125	57	5	307	67	0.619
Kamboh, M. I.^49^	310	111	11	731	133	71	29	6	171	41	0.205
Lambert, J. -C.^30^	428	119	11	975	141	407	168	21	982	210	0.480
Kang, D. E.^13^	127	26	4	280	34	65	34	3	164	40	0.563

HWE: Hardy-Weinberg equilibrium. ^a^ *P* value for HWE test in controls.

### Meta-analysis and meta-regression results

**Figure 2:**
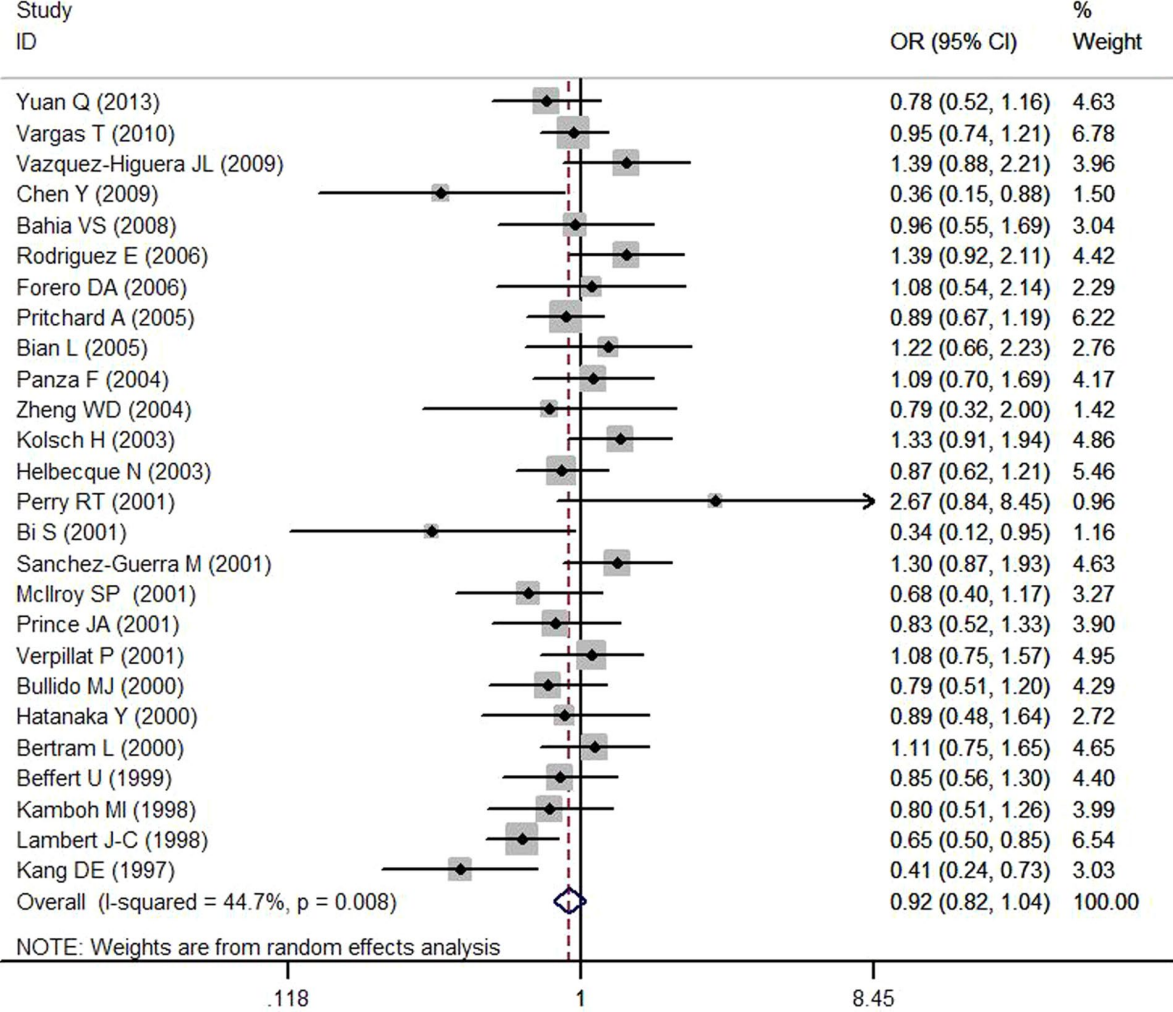
Forest plot of association between LRP1 C766T polymorphism (TT + CT *vs*. CC) and AD susceptibility.

The combined analysis showed that there was no significant association between LRP1 C766T polymorphism and AD susceptibility in any genetic model (T versus C: OR = 0.905, 95% CI = 0.813–1.008, *P* = 0.069; TT versus CC: OR = 0.791, 95% CI = 0.622–1.005, *P* = 0.055; CT versus CC: OR = 0.915, 95% CI = 0.813–1.030, *P* = 0.139; TT + CT versus CC: OR = 0.920, 95% CI = 0.817–1.037, *P* = 0.172; TT versus CC + CT: OR = 0.815, 95% CI = 0.640–1.037, *P* = 0.095) (Table 3 and Fig. 2).

**Table 3:** Meta-analysis of LRP1 C766T polymorphism and AD susceptibility.

Population	Comparison	Sample size		N^a^	Association		Model	Heterogeneity		Publication bias
		AD	Control		OR (95% CI)	*P*		*P*	*I* ^2^ (%)	*P*
Overall	T *vs*. C	6181	6021	25	0.905 (0.813, 1.008)	0.069	Random	0.013	43.0	0.849
	TT *vs*. CC	6074	5943	24	0.791 (0.622, 1.005)	0.055	Fixed	0.623	0	0.971
	CT *vs*. CC	6181	6021	25	0.915 (0.813, 1.030)	0.139	Random	0.031	37.5	0.758
	TT + CT *vs*. CC	6455	6304	26	0.920 (0.817, 1.037)	0.172	Random	0.008	44.7	0.829
	TT *vs*. CC + CT	6074	5943	24	0.815 (0.640, 1.037)	0.095	Fixed	0.683	0	0.972
Caucasian	T *vs*. C	4704	4522	15	0.905 (0.801, 1.022)	0.107	Random	0.019	48.4	0.959
	TT *vs*. CC	4704	4522	15	0.777 (0.595, 1.013)	0.062	Fixed	0.329	11.1	0.901
	CT *vs*. CC	4704	4522	15	0.916 (0.795, 1.055)	0.223	Random	0.021	47.7	0.950
	TT + CT *vs*. CC	4978	4805	16	0.926 (0.806, 1.065)	0.281	Random	0.008	52.3	0.861
	TT *vs*. CC + CT	4704	4522	15	0.799 (0.612, 1.043)	0.099	Fixed	0.353	8.9	0.941
Asian	T *vs*. C	864	1010	6	0.786 (0.635, 0.974)	0.028	Fixed	0.156	37.5	0.460
	TT *vs*. CC	864	1010	6	0.642 (0.297, 1.386)	0.259	Fixed	0.764	0	0.786
	CT *vs*. CC	864	1010	6	0.810 (0.648, 1.011)	0.063	Fixed	0.351	10.1	0.279
	TT + CT *vs*. CC	864	1010	6	0.800 (0.647, 0.990)	0.040	Fixed	0.232	27.0	0.388
	TT *vs*. CC + CT	864	1010	6	0.687 (0.315, 1.498)	0.346	Fixed	0.825	0	0.732
EOAD	T *vs*. C	355	300	3	0.966 (0.743, 1.257)	0.799	Fixed	0.332	9.3	0.977
	TT *vs*. CC	321	267	2	1.506 (0.477, 4.750)	0.485	Fixed	0.719	0	NA
	CT *vs*. CC	355	300	3	0.906 (0.699, 1.174)	0.454	Fixed	0.435	0	0.922
	TT + CT *vs*. CC	355	300	3	0.933 (0.727, 1.198)	0.587	Fixed	0.363	1.2	0.947
	TT *vs*. CC + CT	321	267	2	1.536 (0.484, 4.873)	0.467	Fixed	0.769	0	NA
LOAD	T *vs*. C	1524	1832	10	0.858 (0.748, 0.985)	0.029	Fixed	0.423	1.7	0.346
	TT *vs*. CC	1524	1832	10	0.678 (0.374, 1.229)	0.200	Fixed	0.889	0	0.994
	CT *vs*. CC	1524	1832	10	0.880 (0.767, 1.009)	0.066	Fixed	0.176	29.2	0.702
	TT + CT *vs*. CC	1524	1832	10	0.871 (0.763, 0.994)	0.040	Fixed	0.255	20.4	0.520
	TT *vs*. CC + CT	1524	1832	10	0.714 (0.394, 1.294)	0.267	Fixed	0.875	0	0.861
APOE ε4+	T *vs*. C	924	308	6	0.706 (0.436, 1.145)	0.158	Random	0.051	54.6	0.446
	TT *vs*. CC	815	252	4	0.743 (0.320, 1.723)	0.489	Fixed	0.532	0	0.378
	CT *vs*. CC	924	308	6	0.716 (0.407, 1.257)	0.244	Random	0.048	55.2	0.683
	TT + CT *vs*. CC	1073	363	7	0.790 (0.475, 1.313)	0.363	Random	0.030	57.1	0.683
	TT *vs*. CC + CT	815	252	4	0.770 (0.331, 1.791)	0.544	Fixed	0.528	0	0.369
APOE ε4−	T *vs*. C	819	1207	6	1.054 (0.894, 1.242)	0.530	Fixed	0.591	0	0.546
	TT *vs*. CC	819	1207	6	0.883 (0.475, 1.641)	0.693	Fixed	0.924	0	0.776
	CT *vs*. CC	819	1207	6	1.095 (0.926, 1.295)	0.288	Fixed	0.491	0	0.360
	TT + CT *vs*. CC	944	1435	7	1.120 (0.967, 1.298)	0.130	Fixed	0.403	2.90	0.386
	TT *vs*. CC+CT	819	1207	6	0.876 (0.470, 1.632)	0.677	Fixed	0.924	0	0.665

OR: odds ratio; CI: Confidence interval; EOAD: early onset of AD; LOAD: late onset of AD. ^a^Number of comparisons.

**Figure 3:**
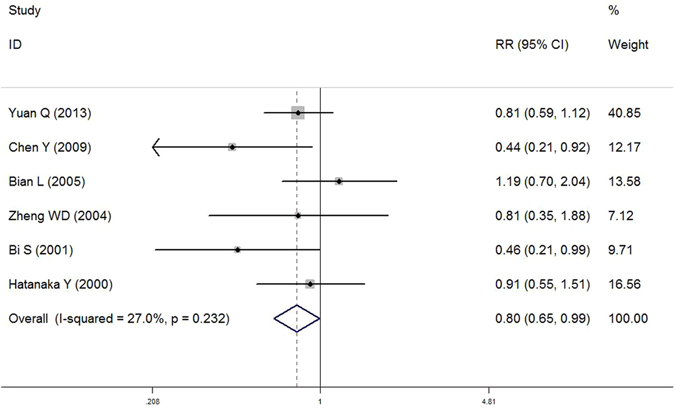
Forest plot of association between LRP1 C766T polymorphism (TT + CT *vs*. CC) and AD susceptibility in Asian population.

**Figure 4:**
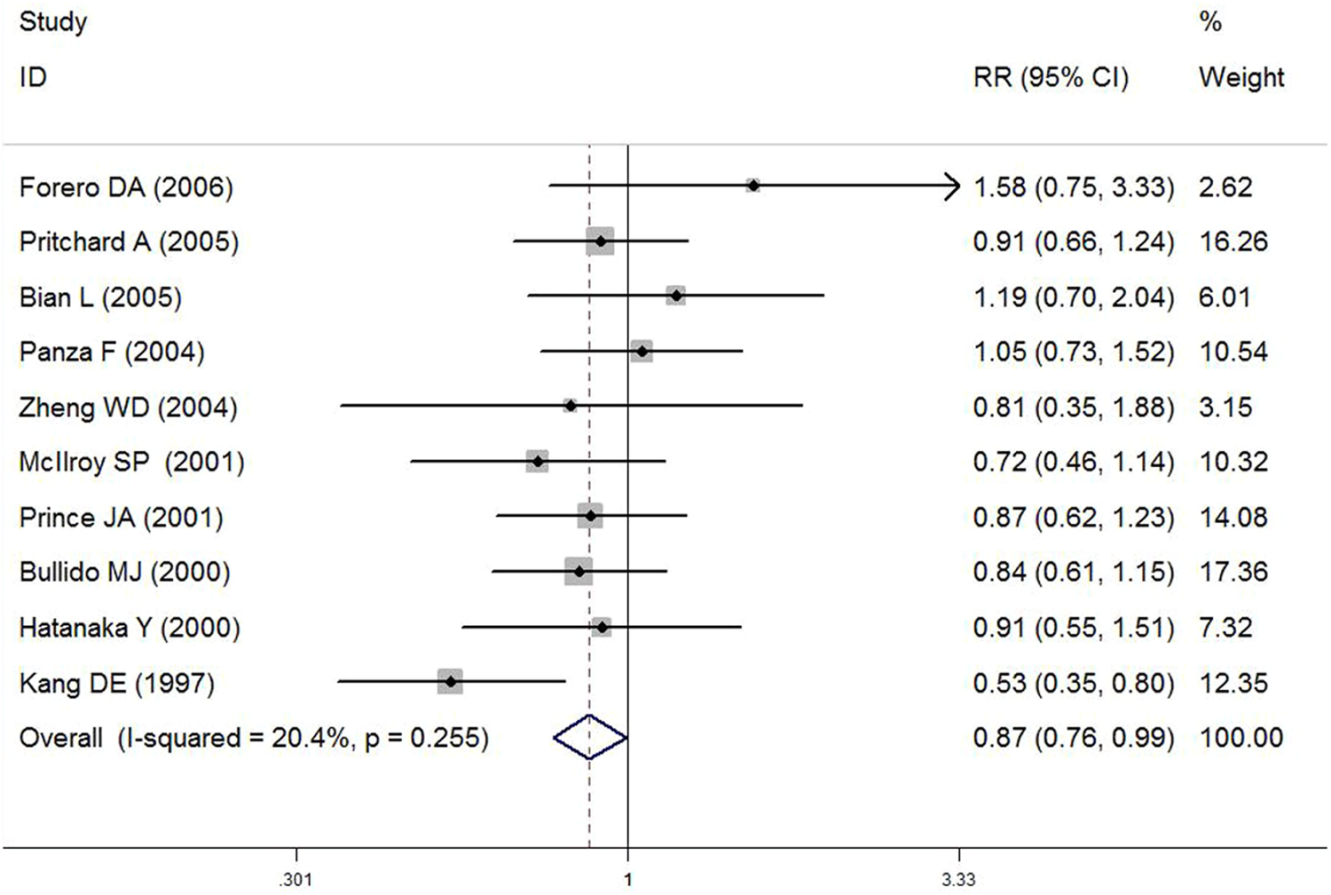
Forest plot of association between LRP1 C766T polymorphism (TT + CT *vs*. CC) and AD susceptibility in LOAD population.

In subgroup analysis by ethnicity, T allele of LRP1 C766T was found to be associated with decreased AD susceptibility among Asian population (T versus C: OR = 0.786, 95% CI = 0.635–0.974, *P* = 0.028; TT + CT versus CC: OR = 0.800, 95% CI = 0.647–0.990, *P* = 0.040) (Fig. 3). However, we did not observe any association for all comparisons in Caucasians. When stratified by time of AD onset, we found T allele of LRP1 C766T may act as a protective factor for late onset of AD (LOAD) (T versus C: OR = 0.858, 95% CI = 0.748–0.985, *P* = 0.029; TT + CT versus CC: OR = 0.871, 95% CI = 0.763–0.994, *P* = 0.040) (Fig. 4), but no significant association was observed for early onset of AD (EOAD). Furthermore, no significant interaction was observed for APOE ε4 status (*P* > 0.05).

The results of univariate and multivariate meta-regression analyses showed that age, MMSE and/or APOE ε4 were not potential factor(s) for heterogeneity among those studies, but gender might contributed to the heterogeneity (as shown in Table 4).

**Table 4:** The potential sources of heterogeneity between LRP1 polymorphism and AD risk were evaluated by both of univariate and multivariate meta-regression analyses.

Heterogeneity factors	Coefficient	95% CI	SE	P
Age				
Univariate	0.008	(−0.027, 0.043)	0.017	0.644
Multivariate	−0.018	(−0.051, 0.015)	0.015	0.251
Gender				
Univariate	1.864	(0.383, 3.345)	0.712	0.016
Multivariate	2.193	(0.233, 4.152)	0.907	0.031
MMSE				
Univariate	−0.081	(−0.344, 0.182)	0.127	0.532
Multivariate	0.004	(−0.268, 0.277)	0.126	0.975
APOE ε4 status				
Univariate	−0.048	(−0.440, 0.343)	0.186	0.798
Multivariate	0.190	(−0.252, 0.632)	0.204	0.37

SE = standard error; 95%CI = 95% confidence interval.

### Publication bias

**Figure 5:**
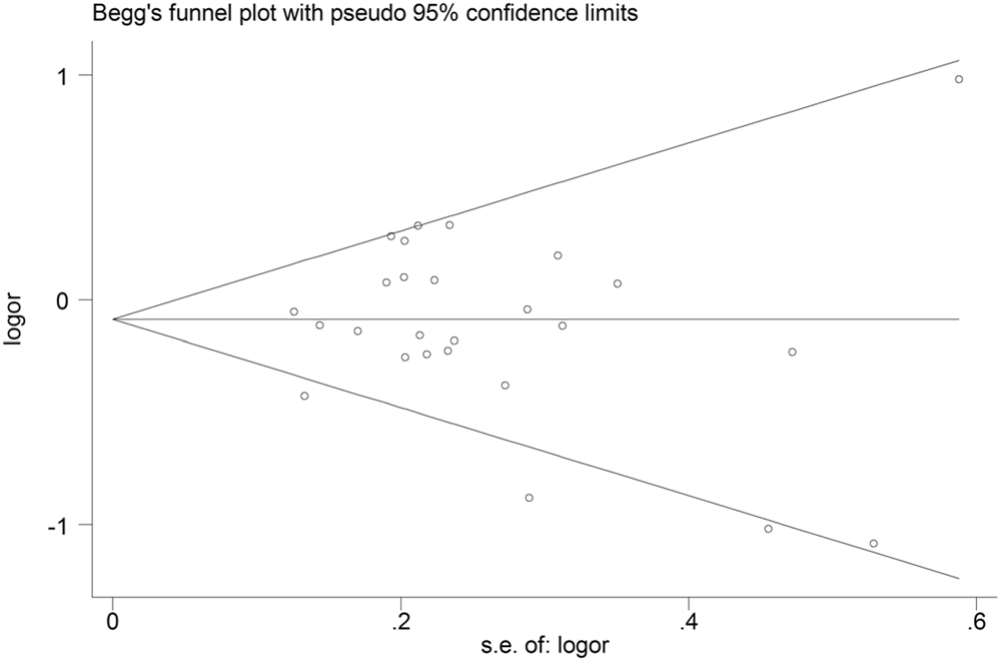
Funnel plot of association between LRP1 C766T polymorphism (TT + CT *vs*. CC) and AD susceptibility.

Begg’s test and Egger’s test were performed to evaluate the publication bias of the included studies. The shape of Begg’s funnel plot appeared to be approximately symmetrical (Fig. 5). Besides, statistical significance was also not observed according to Egger’s test (*P* > 0.05, Table 3). In general, there was no publication bias in our included studies.

## Discussion

AD, as a continuum, bring about serious threat to human health. Considering early detection and intervention at the asymptomatic stage may offer better chance of therapeutic success, it is urgent to identify early diagnostic biomarkers^18, 19^. LRP1, a member of the LDL receptor family, is an endocytic receptor for more than 40 structurally diverse ligands. The findings of previous studies indicate that LRP1 and many of its ligands (eg. APOE and A2M) are co-deposited with Aβ in senile plaques in AD brains^20, 21^. Subsequent studies demonstrated that LRP1 modulates the clearance of Aβ via receptor-mediated pathway in central nervous system^22–24^. Besides, soluble LRP1 provides an endogenous peripheral ‘sink’ activity for Aβ by preventing plasma free Aβ access to the brain^25^. It has also been reported that LRP1 is responsible for a rapid peripheral uptake of Aβ by the liver, which plays a key role in systemic clearance of Aβ^26^. On the other hand, endocytosis of LRP1 could modulate APP trafficking, and contribute to Aβ generation^27^. Interestingly, LRP1 can regulate Aβ metabolism in two contrary sides.

The association between LRP1 polymorphisms and AD susceptibility also has been described extensively, especially exon 3 C766T polymorphism. Kang *et al*. first reported the LRP1 C766T polymorphism, and found a positive association between C allele and AD susceptibility^13^. This finding was replicated in some following studies^28–30^, but Kolsch *et al*. found the opposite result that carriers of a C allele were at lower risk of AD^31^, while some failed to show any association between LRP1 C766T polymorphism and AD^32–45^. Previously, three meta-analysis have tried to clarify the relationship between LRP1 C766T polymorphism and AD susceptibility, which one revealed a weak correlation of LRP1 CC genotype with AD^40^, but other two separately studies showed that no positive evidence was involved in the relationship between this polymorphism and AD risk among overall^36^ and Chinese population^46^. Since several factors could be responsible for these discrepancies, such as inadequate sample size, variability in phenotype definition and allele frequency polymorphisms in different ethnic backgrounds^47^, we conducted a comprehensive meta-analysis with different genetic models in this study, to better clarify the association between LRP1 C766T polymorphism and AD susceptibility.

New results from our research did not show any association of LRP1 C766T polymorphism with AD susceptibility from 6455 AD cases and 6304 controls in overall population. This result is consistent with two published meta-analyses^36, 46^. Compared with the results from previous studies, our data from meta-analysis was relatively reliable to illustrate the association between LRP1 C766T polymorphism and AD susceptibility, because we used different genetic models with a larger number of case-controls.

Due to that people in different ethnic populations may have different allele frequency, and can affect the heterogeneity, we additionally conducted subgroup analysis by ethnicity, time of AD onset and APOE ε4 status. The outcomes by subgroups revealed that T allele of LRP1 C766T could reduce the risk of AD in allele model (T versus C) and dominant model (TT + CT versus CC) among Asian population, no significant role was found in Caucasian group. In terms of onset age, the results from subgroup analysis showed that T allele of LRP1 C766T could act as a protective factor for late onset of AD, but no significant association with early onset of AD. This is also consistent with previous report^13^.

It’s recognized that APOE ε4 is an important pathogenic factor for the development of AD. Several studies have revealed a possible protective effect of TT genotypes in carriers of APOE ε4 alleles^48, 49^. However, APOE ε4 status did not show that the influence of the association between LRP1 C766T polymorphism and AD susceptibility in our study. Moreover, our meta-regression analysis also showed that APOE ε4 status, age, and MMSE were not responsible for heterogeneity.

LRP1 C766T polymorphism is a silent mutation, which does not change the amino acid sequence or splice site. Therefore, it is unlikely to alter the biological function by a direct causal effect with the polymorphism. Some studies consider that the LRP1 C766T polymorphism might be responsible for susceptibility to AD by interact with other genes, such as APOE^48–51^, MAPT^52^, and MAPK8IP1^53^. In addition, some speculated that LRP1 C766T may be in linkage disequilibrium with a deleterious mutation in the LRP1 gene, or with other biologically relevant mutation on neighbouring genes, which affected LRP1 expression^44, 50^. Besides, several studies have a hypothesis that the LRP1 C766T polymorphism might alter the secondary structure of the LRP mRNA to affect the translation and stability of the protein^13, 48^. To date, the conclusion with LRP1 C766T polymorphism with AD susceptibility is conflicting, further genetic analyses of this locus are needed to illuminate the potential mechanism and the functional interactions with AD.

Some limitations of our meta-analysis should be acknowledged. The sample size in some subgroup analysis was small, which may increase the risk of false negatives or false positives. Besides, we did not perform subgroup analysis based on other factors participated in the progression of AD, such as educational background, due to a lack of sufficient information. Larger and broader independent investigations are required to better understand the role of LRP1 C766T polymorphism in AD pathogenesis.

In conclusion, our meta-analysis suggested that LRP1 C766T polymorphism was associated with lower risk of AD in Asian, and could reduce LOAD risk especially. Furthermore, large-scale studies should be performed to reach more understanding of this association.

## Materials and Methods

### Search strategy

We searched electronic databases PubMed, Embase and CNKI (up to August 2016) using the following keywords: (“Alzheimer’s disease” or “Alzheimer disease” or “AD”) and (“low density lipoprotein receptor-related protein 1” or “LDL receptor-related protein 1” or “LRP1”) and (“polymorphism” or “SNP” or “variant” or “genotype”) without language restriction. The bibliographies of the retrieved studies were also screened to identify relevant publications.

### Inclusion and exclusion criteria

The eligible studies had to meet all the following criteria: (1) a case–control study to evaluate the association between LRP1 C766T polymorphism and risk of AD; (2) useful data including sample size, allele or genotype distribution were given; (3) genotype distribution of controls followed the HWE. Accordingly, the exclusion criteria were as follows: (1) reviews, meta-analysis or editorial articles; (2) studies were provided with inadequate information; (3) for the studies with overlapping data, only the most relevant articles with the largest dataset were included in the final analysis.

The literature retrieval and inclusion were carried out in duplication by two independent reviewers.

### Data extraction

Two reviewers independently extracted the following information: first author, year of publication, country, ethnicity, total number of cases and controls, mean age of cases and controls, proportion of female in cases and controls, AD diagnosis criteria, genotyping method, source of controls, time of AD onset, genotype or/and allele distribution in cases and controls. If conflicting results produced, two reviewers would review the publications again and reached a consensus by discussion.

### Quality assessment

Two reviewers independently assessed the quality of each included studies in the meta-analysis according to the criteria of quality assessment (as referred in the Reference of ^54, 55^), and the disagreements were judged by the third reviewer to ensure a consistent outcome. Quality scores of studies ranged from 0 (the lowest) to 15 (the highest). Studies with quality scores among 10 to 15 were grouped into high quality studies and other studies scored between 0 and 9 were categorized into low quality studies.

### Statistical analysis

HWE in controls was tested by a chi-square test. Summary odds ratio (OR) with confidence interval (95% CI) for genotypes and alleles were used to evaluate the strength of association between LRP1 C766T polymorphism and AD susceptibility. The significance of the pooled OR was measured using the *Z*-test. Four genetic models were performed in our meta-analysis: allele model (T versus C), codominant model [homozygote comparison (TT versus CC) and heterozygote comparison (CT versus CC)], dominant model (TT + CT versus CC), and recessive model (TT versus CC + CT). The heterogeneity was also quantified with *I* ^2^ statistics. If no significant heterogeneity was found between the studies, the pooled OR was calculated by using the fixed effects model (the Mantel-Haenszel method)^56^. Otherwise, the random effects model (the DerSimonian and Laird method) was applied^57^. Both of univariate and multivariate meta-regression analyses were also carried out to explore potential sources of heterogeneity among studies. The log of the ORs from involved studies was using as dependent variables, and age, gender, Mini-Mental State Exam (MMSE) and/or APOE ε4 status as covariates. Publication bias was tested by Begg’s test and Egger’s test^58, 59^. We also performed subgroup analysis according to ethnicity, time of AD onset and APOE ε4 status, respectively. Statistical analyses were conducted with Stata Version 11.0 (College Station, TX, USA), and a two-sided *P* < 0.05 was considered statistically significant.

## References

[1] 2016 Alzheimer’s disease facts and figures. *Alzheimer’s & Dementia* **12**, 459–509 (2016). 10.1016/j.jalz.2016.03.001 27570871

[2] Dartigues JF (2009). Alzheimer’s disease: a global challenge for the 21st century. The Lancet Neurology.

[3] Bettens K, Sleegers K, Van Broeckhoven C (2013). Genetic insights in Alzheimer’s disease. The Lancet Neurology.

[4] Farrer LA (2015). Expanding the genomic roadmap of Alzheimer’s disease. The Lancet Neurology.

[5] Gaiteri C, Mostafavi S, Honey CJ, De Jager PL, Bennett DA (2016). Genetic variants in Alzheimer disease - molecular and brain network approaches. Nature reviews. Neurology.

[6] Sagare AP, Deane R, Zlokovic BV (2012). Low-density lipoprotein receptor-related protein 1: a physiological Abeta homeostatic mechanism with multiple therapeutic opportunities. Pharmacology & therapeutics.

[7] Lillis AP, Van Duyn LB, Murphy-Ullrich JE, Strickland DK (2008). LDL receptor-related protein 1: unique tissue-specific functions revealed by selective gene knockout studies. Physiological reviews.

[8] Zlokovic BV, Deane R, Sagare AP, Bell RD, Winkler EA (2010). Low-density lipoprotein receptor-related protein-1: a serial clearance homeostatic mechanism controlling Alzheimer’s amyloid beta-peptide elimination from the brain. Journal of neurochemistry.

[9] Boucher P, Herz J (2011). Signaling through LRP1: Protection from atherosclerosis and beyond. Biochemical pharmacology.

[10] Liu CC (2015). Neuronal LRP1 regulates glucose metabolism and insulin signaling in the brain. The Journal of neuroscience: the official journal of the Society for Neuroscience.

[11] Tian X (2015). LRP-1-mediated intracellular antibody delivery to the Central Nervous System. Scientific reports.

[12] Kanekiyo T, Bu G (2014). The low-density lipoprotein receptor-related protein 1 and amyloid-beta clearance in Alzheimer’s disease. Frontiers in aging neuroscience.

[13] Kang DE (1997). Genetic association of the low-density lipoprotein receptor-related protein gene (LRP), an apolipoprotein E receptor, with late-onset Alzheimer’s disease. Neurology.

[14] Hollenbach E, Ackermann S, Hyman BT, Rebeck GW (1998). Confirmation of an association between a polymorphism in exon 3 of the low-density lipoprotein receptor-related protein gene and Alzheimer’s disease. Neurology.

[15] Zhou YT (2008). Genetic association between low-density lipoprotein receptor-related protein gene polymorphisms and Alzheimer’s disease in Chinese Han population. Neuroscience letters.

[16] Zhou XH, Yue YH, Miao HJ, Hong Y, Ka-Bi N (2008). Association of the low density lipoprotein receptor-related protein gene 766C/T polymorphism with Alzheimer’s disease in Xinjiang Uygurs and Hans. Zhonghua yi xue yi chuan xue za zhi = Zhonghua yixue yichuanxue zazhi = Chinese journal of medical genetics.

[17] Feng YQ (2006). Correlation of the polymorphisms of apolipoprotein E gene and low-density lipoprotein receptor related protein gene with sporadic Alzheimer’s disease. Journal of International Neurology and Neuosurgery.

[18] Chase A (2014). Alzheimer disease: advances in imaging of AD biomarkers could aid early diagnosis. Nature reviews. Neurology.

[19] Dubois B (2016). Preclinical Alzheimer’s disease: Definition, natural history, and diagnostic criteria. Alzheimer’s & dementia: the journal of the Alzheimer’s Association.

[20] Namba Y, Tomonaga M, Kawasaki H, Otomo E, Ikeda K (1991). Apolipoprotein E immunoreactivity in cerebral amyloid deposits and neurofibrillary tangles in Alzheimer’s disease and kuru plaque amyloid in Creutzfeldt-Jakob disease. Brain research.

[21] Rebeck GW, Harr SD, Strickland DK, Hyman BT (1995). Multiple, diverse senile plaque-associated proteins are ligands of an apolipoprotein E receptor, the alpha 2-macroglobulin receptor/low-density-lipoprotein receptor-related protein. Annals of neurology.

[22] Arelin K (2002). LRP and senile plaques in Alzheimer’s disease: colocalization with apolipoprotein E and with activated astrocytes. Brain research. Molecular brain research.

[23] Kang DE (2000). Modulation of amyloid beta-protein clearance and Alzheimer’s disease susceptibility by the LDL receptor-related protein pathway. The Journal of clinical investigation.

[24] Kanekiyo T (2013). Neuronal clearance of amyloid-beta by endocytic receptor LRP1. The Journal of neuroscience: the official journal of the Society for Neuroscience.

[25] Sagare A (2007). Clearance of amyloid-beta by circulating lipoprotein receptors. Nature medicine.

[26] Tamaki C (2006). Major involvement of low-density lipoprotein receptor-related protein 1 in the clearance of plasma free amyloid beta-peptide by the liver. Pharmaceutical research.

[27] Cam JA, Zerbinatti CV, Li Y, Bu G (2005). Rapid endocytosis of the low density lipoprotein receptor-related protein modulates cell surface distribution and processing of the beta-amyloid precursor protein. The Journal of biological chemistry.

[28] Bi S, Zhang Y, Wu J, Wang D, Zhao Q (2001). Association between low-density lipoprotein receptor-related protein gene, butyrylcholinesterase gene and Alzheimer’s disease in Chinese. Chinese medical sciences journal = Chung-kuo i hsueh k’o hsueh tsa chih.

[29] Chen Y, Zhang SL, Yue Y (2009). Relationship between the polymorphism of low-density lipoprotein receptor-related protein gene, butyrylcholinesterase-K variant and Alzheimer’s disease. Practical Geriatrics.

[30] Lambert J-C, Vrièze FW-D, Amouyel P, Chartier-Harlin M-C (1998). Association at LRP gene locus with sporadic late-onset Alzheimer’s disease. The Lancet.

[31] Kolsch H (2003). Association of the C766T polymorphism of the low-density lipoprotein receptor-related protein gene with Alzheimer’s disease. American journal of medical genetics. Part B, Neuropsychiatric genetics: the official publication of the International Society of Psychiatric Genetics.

[32] Vargas T (2010). A megalin polymorphism associated with promoter activity and Alzheimer’s disease risk. American journal of medical genetics. Part B, Neuropsychiatric genetics: the official publication of the International Society of Psychiatric Genetics.

[33] Bahia VS (2008). Polymorphisms of APOE and LRP genes in Brazilian individuals with Alzheimer disease. Alzheimer disease and associated disorders.

[34] Rodriguez E (2006). Genetic interaction between two apolipoprotein E receptors increases Alzheimer’s disease risk. Journal of neurology.

[35] Forero DA, Arboleda G, Yunis JJ, Pardo R, Arboleda H (2006). Association study of polymorphisms in LRP1, tau and 5-HTT genes and Alzheimer’s disease in a sample of Colombian patients. Journal of neural transmission.

[36] Pritchard A (2005). Association study and meta-analysis of low-density lipoprotein receptor related protein in Alzheimer’s disease. Neuroscience letters.

[37] Panza F (2004). Regional European differences in allele and genotype frequencies of low density lipoprotein receptor-related protein 1 polymorphism in Alzheimer’s disease. American journal of medical genetics. Part B, Neuropsychiatric genetics: the official publication of the International Society of Psychiatric Genetics.

[38] Zheng, W. D. *et al*. A genetic association study between the cardiovascular risk factor and late-onset Alzheimer Disease in Guangxi Han Chinese, *Chinese Journal of Neuroimmunology and Neurology* **11**, 68–71+90 (2004).

[39] Perry RT, Collins JS, Harrell LE, Acton RT, Go RC (2001). Investigation of association of 13 polymorphisms in eight genes in southeastern African American Alzheimer disease patients as compared to age-matched controls. American journal of medical genetics.

[40] Sanchez-Guerra M (2001). Case-control study and meta-analysis of low density lipoprotein receptor-related protein gene exon 3 polymorphism in Alzheimer’s disease. Neuroscience letters.

[41] McIlroy SP (2001). Common polymorphisms in LRP and A2M do not affect genetic risk for Alzheimer disease in Northern Ireland. American journal of medical genetics.

[42] Prince JA (2001). Lack of replication of association findings in complex disease: an analysis of 15 polymorphisms in prior candidate genes for sporadic Alzheimer’s disease. European journal of human genetics: EJHG.

[43] Verpillat P (2001). Use of haplotype information to test involvement of the LRP gene in Alzheimer’s disease in the French population. European journal of human genetics: EJHG.

[44] Beffert U, Arguin C, Poirier J (1999). The polymorphism in exon 3 of the low density lipoprotein receptor-related protein gene is weakly associated with Alzheimer’s disease. Neuroscience letters.

[45] Bertram L (2000). Candidate genes showing no evidence for association or linkage with Alzheimer’s disease using family-based methodologies. Experimental gerontology.

[46] Yang L (2015). Association of PS1 1/2, ACE I/D, and LRP C/T polymorphisms with Alzheimer’s disease in the Chinese population: a meta-analysis of case-control studies. Genetics and molecular research: GMR.

[47] Greene CS, Penrod NM, Williams SM, Moore JH (2009). Failure to replicate a genetic association may provide important clues about genetic architecture. PloS one.

[48] Hatanaka Y (2000). Low density lipoprotein receptor-related protein gene polymorphisms and risk for late-onset Alzheimer’s disease in a Japanese population. Clinical genetics.

[49] Kamboh MI, Ferrell RE, DeKosky ST (1998). Genetic association studies between Alzheimer’s disease and two polymorphisms in the low density lipoprotein receptor-related protein gene. Neuroscience letters.

[50] Yuan Q, Wang F, Xue S, Jia J (2013). Association of polymorphisms in the LRP1 and A2M genes with Alzheimer’s disease in the northern Chinese Han population. Journal of clinical neuroscience: official journal of the Neurosurgical Society of Australasia.

[51] Bullido MJ (2000). Alzheimer’s risk associated with human apolipoprotein E, alpha-2 macroglobulin and lipoprotein receptor related protein polymorphisms: absence of genetic interactions, and modulation by gender. Neuroscience letters.

[52] Vazquez-Higuera JL (2009). Genetic interaction between tau and the apolipoprotein E receptor LRP1 Increases Alzheimer’s disease risk. Dementia and geriatric cognitive disorders.

[53] Helbecque N (2003). Islet-brain1/C-Jun N-terminal kinase interacting protein-1 (IB1/JIP-1) promoter variant is associated with Alzheimer’s disease. Molecular psychiatry.

[54] Liu S, Zeng F, Wang C (2015). The nitric oxide synthase 3 G894T polymorphism associated with Alzheimer’s disease risk: a meta-analysis. Scientific Reports.

[55] He J, Liao XY, Zhu JH (2014). Association of MTHFR C677T and A1298C polymorphisms with non-Hodgkin lymphoma susceptibility: evidence from a meta-analysis. Scientific Reports.

[56] Mantel N, Haenszel W (1959). Statistical aspects of the analysis of data from retrospective studies of disease. Journal of the National Cancer Institute.

[57] DerSimonian R, Kacker R (2007). Random-effects model for meta-analysis of clinical trials: an update. Contemporary clinical trials.

[58] Begg CB, Mazumdar M (1994). Operating characteristics of a rank correlation test for publication bias. Biometrics.

[59] Egger M, Davey Smith G, Schneider M, Minder C (1997). Bias in meta-analysis detected by a simple, graphical test. BMJ (Clinical research ed.).

[60] Bian L (2005). Association study of the A2M and LRP1 Genes with Alzheimer disease in the Han Chinese. Biological psychiatry.

